# Response: Commentary: Is So-Called “Split Alpha” in EEG Spectral Analysis a Result of Methodological and Interpretation Errors?

**DOI:** 10.3389/fnins.2021.784338

**Published:** 2021-11-11

**Authors:** Ewa Zalewska

**Affiliations:** Nalecz Institute of Biocybernetics and Biomedical Engineering, Polish Academy of Sciences, Warsaw, Poland

**Keywords:** electroencephalography, spectral analysis, Fast Fourier Transform, spurious peaks, Gibbs phenomenon

My comment is short because it is written just to communicate that the Commentary of Olejarczyk and Sobieszek (2021) does not refer to the content of my article Zalewska (2020), but repeats their own erroneous statements from their original article (Olejarczyk et al., 2017). This clearly proves that the Authors did not consider my explanation of their errors and have not used any of what I have presented in publication Zalewska (2020). In this context, both my name and the title of my article Zalewska (2020) are used in an unauthorized manner. The Authors attribute me statements which I have not made, and which is in fact contrary to what I have described in Zalewska (2020).

In what follows, I only shortly point out the inadequate citation of my article by Olejarczyk and Sobieszek (2021) in the Commentary in question.

1. The presented commentary does not refer to my article titled “Is So Called ‘Split Alpha' in EEG Spectral Analysis a Result of Methodological and Interpretation Errors?” (Zalewska, 2020), but rather to the original paper of these Authors (Olejarczyk et al., 2017).

The Commentary, while it pretends to be polemic, does not even attempt any discussion with Zalewska (2020). The paper has no relation to the nature and origin of double alpha effect, so-called “split alpha,” as discussed in Zalewska (2020). Instead, the Authors have written the Commentary describing again the methods they used in the original paper (Olejarczyk et al., 2017).

**My position on this issue:**

The Commentary has not addressed to my article Zalewska (2020).

2. The Authors attribute me statement which I have not made, and which is in fact contrary to what I have described in Zalewska (2020). They need that to try to promote their opinion.

In Section 4, the Authors manipulate what was stated in Zalewska (2020) in an attempt to prove their opinion.

Please compare the Authors' statement:

“*Since the frequency difference between two maxima in the split alpha was 1.4 Hz, in each of these cases the frequency resolution was sufficient to say that this is not a spectrum leakage but the split alpha effect. These results are in accordance with the conclusions formulated by Zalewska (Zalewska*, *2020**).”*

with what was stated in the paper of Zalewska (2020). In Zalewska (2020), it was clearly shown that under the conditions that the Authors mention, it is possible to have two peaks separated by 5 Hz emerge due to the Gibbs phenomenon. In fact, double peaks may appear at the edges of a frequency band, whether the band is 1.4 Hz wide or 5 Hz wide. But contrary to what the Authors think, the argument that the peak separation of 1.4 Hz is larger than the resolution does not imply that the peaks are real. This is what I have shown, and I have provided arguments how to verify that. The Authors have not used any of what I have presented. From the spectra presented in their original paper (Olejarczyk et al., 2017), it may be clearly seen that the peaks are superposed on a band of frequencies which is a telltale of a possible Gibbs phenomenon.

The Authors do not provide exact citation of the section of Zalewska (2020) which, according to them, supports their opinion. I strongly state that what they do write is, in view of Zalewska (2020), false. The reference to my paper is unauthorized. I do not want to be cited as seemingly supporting the Authors' misconceptions.

**My position on this issue:**

The Authors have no right to state that Zalewska has shown what they state.

3. In fact, the second reference (last sentence of Section 4 in the Commentary) to Zalewska (2020) also does not relate to the Gibbs phenomenon and to my paper.

The reason for this request is that while the Authors cite in their paper multiple methods, they present results based on FFT; thus, the other methods do not serve them in any way to elucidate the underlying phenomenon responsible for the double alpha effect. Thus, their claim:

“*However, the main purpose of this commentary was to explain better the methodological issues related to the FFT application discussed in the publication of Zalewska (Zalewska*, *2020**).”*

is in no way corroborated by what they write in Section 4 (or in fact in the whole Commentary). The Authors again try to use flawed logic because what their statement means is that they imply that they have shown that FFT is not applicable by using the other methods mentioned by them. But they present results for FFT. So, by the same token it may be concluded that their EEG spectra based on FFT are incorrect. They miss the point—since it was the FFT spectra that exhibit double alpha, then explanation should be sought in terms of signal theory which they do not attempt to do. They try to use other methods to find the underlying elusive brain physics which they fail. On the contrary, I have shown how the double alpha can be explained by the well-known Gibbs phenomenon. So, in effect what they write is in no relation to what Zalewska (2020) has done and hence the reference is unauthorized. Again, the Authors are free to write what they choose; however, adding random references to their statements should not be a part of a regular scientific method.

**My position on this issue:**

The Authors do not “*explain better the methodological issues related to the FFT application discussed in the publication of Zalewska (Zalewska*, *2020**)”* at all. What is the most important is that the Authors do not relate to the Gibbs phenomenon, which is the most important issue in my article.

4. My general comment

The Authors fail to distinguish two things: the fact that double peaks can be observed in the FFT spectra of EEG from the reasons for the emergence of these double peaks. Zalewska (2020) in no place makes any claims that the double peaks do not appear in the FFT spectra of EEG; however, contrary to the Authors, Zalewska does provide an explanation in terms of the well-known Gibbs phenomenon. The Authors do not even mention the Gibbs phenomenon in the Commentary (except reference).

I can understand that the Authors were inspired by my paper, but they do not show in any way that they have made use of any of the findings reported by me and restrict their focus to a simple recapitulation of the methods they have used in their original paper. This does not justify including me in their new paper, i.e., Commentary.

The Authors refer to my article in a false way (misinterpretation) while in fact in the Commentary they provide only a comment to their own paper (Olejarczyk et al., 2017). I do not agree to the Authors' trying to support their misconceptions by attributing me statements I have not made in my paper.

The Author's commentary misleads the readers of the Journal, again.

## Author Contributions

The author confirms being the sole contributor of this work and has approved it for publication.

## Conflict of Interest

The author declares that the research was conducted in the absence of any commercial or financial relationships that could be construed as a potential conflict of interest.

## Publisher's Note

All claims expressed in this article are solely those of the authors and do not necessarily represent those of their affiliated organizations, or those of the publisher, the editors and the reviewers. Any product that may be evaluated in this article, or claim that may be made by its manufacturer, is not guaranteed or endorsed by the publisher.
